# Population-Level Impact of Shorter-Course Regimens for Tuberculosis: A Model-Based Analysis

**DOI:** 10.1371/journal.pone.0096389

**Published:** 2014-05-09

**Authors:** Mariam O. Fofana, Gwenan M. Knight, Gabriela B. Gomez, Richard G. White, David W. Dowdy

**Affiliations:** 1 Department of Epidemiology, Johns Hopkins Bloomberg School of Public Health, Baltimore, Maryland, United States of America; 2 Medical Scientist Training Program, Johns Hopkins School of Medicine, Baltimore, Maryland, United States of America; 3 TB Modelling Group, TB Centre and Centre for the Mathematical Modelling of Infectious Diseases, Faculty of Epidemiology and Population Health, London School of Hygiene and Tropical Medicine, London, United Kingdom; 4 Department of Global Health, University of Amsterdam, Amsterdam, The Netherlands; 5 Amsterdam Institute for Global Health and Development, Amsterdam, The Netherlands; 6 Center for Tuberculosis Research, Johns Hopkins University School of Medicine, Baltimore, Maryland, United States of America; Arizona State University, United States of America

## Abstract

Despite current control efforts, global tuberculosis (TB) incidence is decreasing slowly. New regimens that can shorten treatment hold promise for improving treatment completion and success, but their impact on population-level transmission remains unclear. Earlier models projected that a four-month regimen could reduce TB incidence by 10% but assumed that an entire course of therapy must be completed to derive any benefit. We constructed a dynamic transmission model of TB disease calibrated to global estimates of incidence, prevalence, mortality, and treatment success. To account for the efficacy of partial treatment, we used data from clinical trials of early short-course regimens to estimate relapse rates among TB patients who completed one-third, one-half, two-thirds, and all of their first-line treatment regimens. We projected population-level incidence and mortality over 10 years, comparing standard six-month therapy to hypothetical shorter-course regimens with equivalent treatment success but fewer defaults. The impact of hypothetical four-month regimens on TB incidence after 10 years was smaller than estimated in previous modeling analyses (1.9% [95% uncertainty range 0.6–3.1%] vs. 10%). Impact on TB mortality was larger (3.5% at 10 years) but still modest. Transmission impact was most sensitive to the proportion of patients completing therapy: four-month therapy led to greater incidence reductions in settings where 25% of patients leave care (“default”) over six months. Our findings remained robust under one-way variation of model parameters. These findings suggest that novel regimens that shorten treatment duration may have only a modest effect on TB transmission except in settings of very low treatment completion.

## Introduction

Tuberculosis (TB) is the second leading cause of death from a single infectious agent: it is estimated that one-third of the world population is infected with TB, with 8.7 million developing active disease and 1.4 million dying each year [1]. In the last 25 years, over 20 new drugs to treat human immunodeficiency virus (HIV) infection have been developed; by contrast, the primary first-line treatment for TB–requiring six months of therapy with moderately toxic agents–has remained unchanged [2]–[5]. Globally, approximately 7% of TB patients who receive first-line therapy do not complete this six-month course [1], but in some settings this percentage is as high as 30–50% [6]. Incomplete treatment results in higher risk of relapse, continued disease transmission, and emergence of drug resistance [6]. If the goal of global elimination of TB by 2050 is to be attained, it is widely recognized that new drugs capable of curing TB more rapidly will be necessary [1],[7].

For the first time in decades, novel treatment regimens hold the realistic promise of shortening the standard six-month first-line TB treatment course [8]–[10]. If their efficacy is confirmed in ongoing trials, these novel regimens could reduce healthcare costs [11] and improve both patient satisfaction and treatment outcomes [12],[13]. However, a key consideration for public health programs is the potential of novel TB regimens to impact population-level epidemiological outcomes, specifically future incidence and mortality. The expectation that shorter treatment will help control transmission has been a key driver of ongoing efforts by global organizations to develop new drugs and regimens for TB [14],[15].

**Figure 1:**
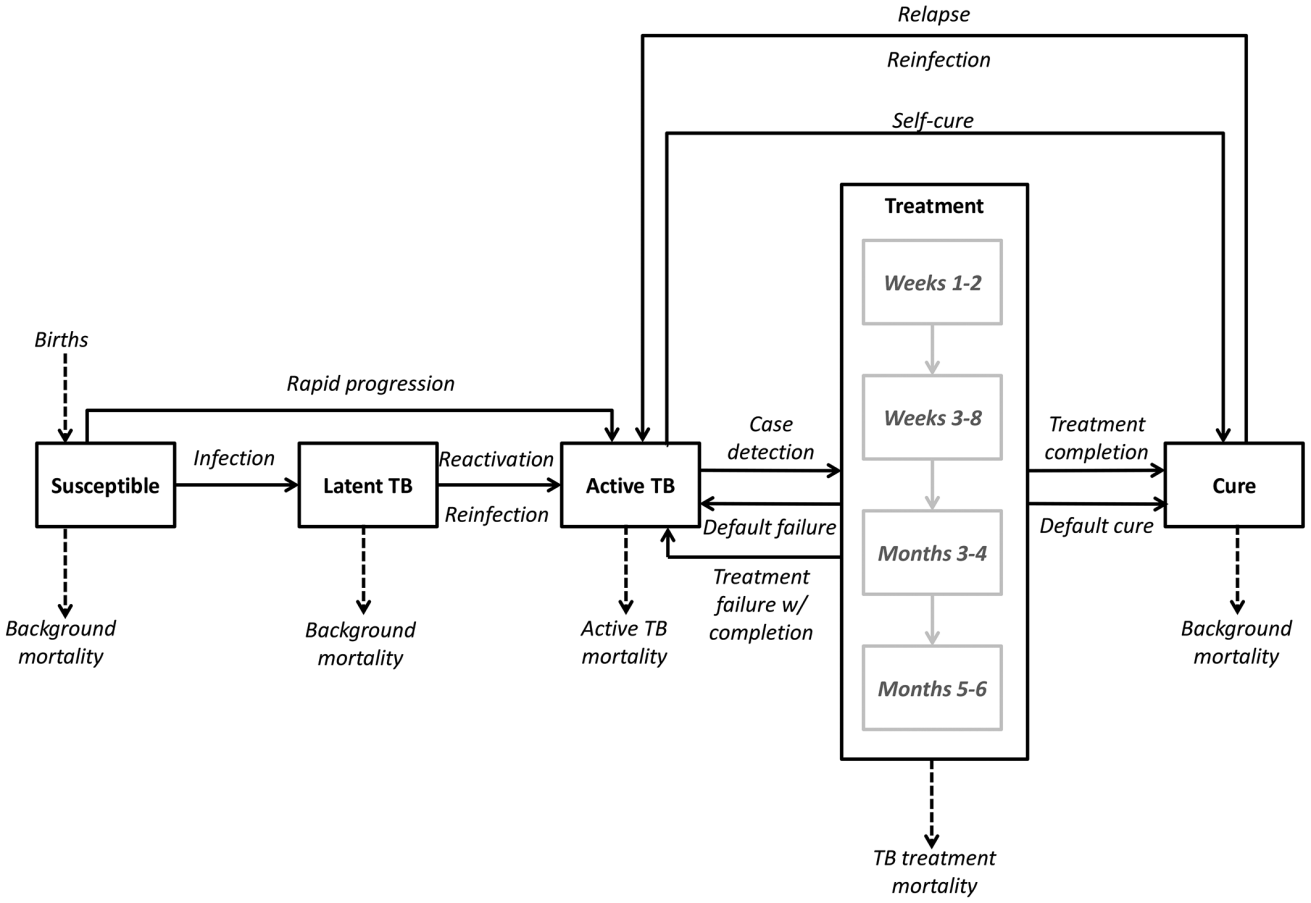
Model compartments and transition rates. Boxes represent the proportions of the modeled population that are susceptible to infection, latently infected with *M. tuberculosis*, in active TB disease, under treatment, or cured. Arrows represent the transitions between various states, including up to four sequential phases of treatment. Rates of transition are described in the Methods section and Appendix S1.

Mathematical (transmission) models are important tools for estimating the potential impact of new technologies and informing policy [16]. Prior models have projected long-term TB incidence reductions of 10–40% from the introduction of shorter-course TB regimens [18]. However, these models have generally assumed that TB therapy is ineffective unless a full course is completed. In reality, patients who receive no treatment can experience spontaneous resolution [19], and follow-up from early randomized trials demonstrates that partial courses of treatment (two to four months) can achieve durable cure in a considerable proportion of patients [20]–[22]. Using data from these trials, we constructed a mathematical model of TB treatment (Figure 1) to more realistically assess the impact of novel, shorter-course first-line treatment regimens (four months, two months, and two weeks) on population-level transmission and compare our results to previous estimates.

## Methods

### Model Structure

We used ordinary differential equations to construct a deterministic compartmental model of TB transmission (Figure 1). This model resembles previous TB models [23],[24] in its basic design but adds additional structure to reflect the process of TB treatment.

Specifically, we model TB treatment as consisting of four sequential phases: weeks 1–2, weeks 3–8, months 3–4, and months 5–6. Individuals with active TB must be successfully diagnosed before they can initiate the first phase of treatment. Upon starting treatment, the bacillary burden decreases rapidly, and individuals on treatment are assumed to be non-infectious after the first two weeks [25],[26]. In each treatment phase, individuals may either die, leave care (“default”), or progress to the next phase (Table 1). Patients who default either return to the active (infectious) state or advance to the “cured/recovered” state; the probability of cure increases with increasing duration of therapy, as informed by data from clinical trials of two-month and four-month treatment regimens [20]–[22]. We took the conservative stance that all individuals who relapse within the longest follow-up period from any available trial (60 months) receive no benefit from treatment and thus return immediately to the active TB compartment; all other individuals are assumed to be cured. Thus, for example, the proportion cured among individuals taking more than four, but less than six, months of standard therapy was set equal to the proportion of individuals who completed a four-month regimen of streptomycin, isoniazid, rifampin, and pyrazinamide and had no long-term relapse. These individuals–like all others who are latently infected or cured (therapeutically or spontaneously)–remain susceptible to reinfection.

**Table 1:** Model inputs for TB treatment outcomes, by treatment phase.

Outcome	Treatment phase					Reference(s)
	*Week 0–2*	*Week 3–8*	*Month 3–4*	*Month 5–6*	*Total*	
**Duration**	2 weeks	6 weeks	2 months	2 months	2 weeks-6 months	
**Percentage defaulting (sensitivity analysis range)**	0.2% (0–1.0%)	1.9% (0–4.1%)	2.7% (0–5.7%)	2.2% (0–4.8%)	7.0% (2–15%)	[1],[6]
**Percentage dying (sensitivity analysis range)**	1.1% (0.5–2.1%)	1.3% (0.6–2.5%)	0.8% (0.4–1.7%)	0.8% (0.4–1.7%)	4.0%	[1],[28]–[30]
**Percentage completing treatment period**	98.7%	96.8%	96.5%	96.9%	-	
**Cumulative percentage remaining in therapy**	98.7%	95.0%	92.1%	89.0%	89.0%	

### Treatment scenarios

**Figure 2:**
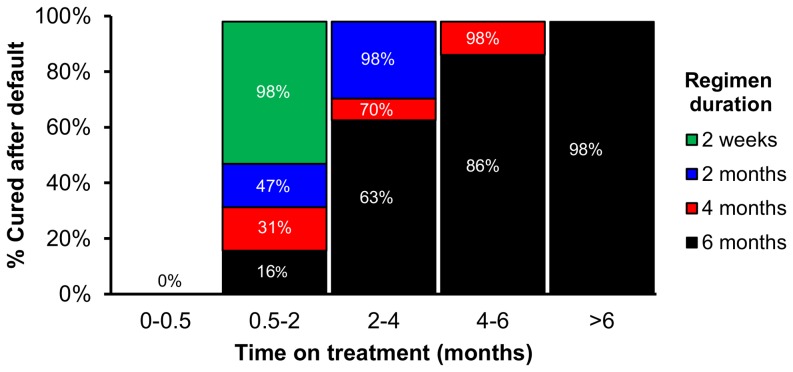
Proportion cured after default, by treatment phase and regimen duration. The proportion cured after default in a six-month treatment regimen was based on outcomes of early TB treatment clinical trials. For each hypothetical shortened treatment regimen, the proportion cured after default is increased according to the proportion of the total treatment duration completed. Detailed examples of calculations are provided in Appendix S1.

Our primary outcomes were TB incidence and mortality at 10 years, comparing continued use of the current six-month regimen to the introduction of novel, shorter regimens (four months, two months and two weeks), assuming that these shorter regimens will have the same efficacy as the current regimen. We defined treatment efficacy as the proportion of people completing the full course of TB therapy who are cured without long-term relapse. Since efficacy is assumed to be similar for all regimens, shorter regimens are modeled as superior to standard therapy in three ways. First, the proportion of treatment completion is higher; for example, any individual who defaults during months 5–6 of a six-month regimen would have completed therapy on a four-month regimen. Second, completion of any treatment phase represents completion of a greater proportion of total treatment in shorter-course regimens, and we model the probability of cure as a function of the proportion of total treatment course completed (beyond the first two weeks). Thus, for example, taking two months of treatment equates to 33% completion of the six-month regimen but 50% completion of a four-month regimen. Probabilities of cure at each phase of treatment are shown in Figure 2. Third, in addition to improving cure rates among those completing therapy, we assume that shorter regimens avert TB-related mortality that otherwise occurs during stages of treatment after the shorter regimen is completed – though this effect may not be large enough to result in statistically superior outcomes in a clinical trial.

### Model assumptions, calibration and data inputs

The model was designed to be simple and transparent, in order to increase the interpretability of results and comparability with previous models of shortened treatment duration. We modeled a hypothetical, non-age-structured population with a life expectancy of 70 years, assuming no net migration or population growth. We excluded non-pulmonary TB, as such cases are unlikely to be infectious and constitute only 14% of notified cases worldwide [1]. Although poor treatment adherence may lead to primary drug resistance, our focus was on first-line regimens, so we did not separately model the transmission of drug-resistant TB. There is no evidence that novel treatment regimens would have differential indications or impact according to HIV status; we therefore modeled our population to reflect the weighted average of WHO-reported outcomes (including both HIV-associated and non-HIV-associated TB). As our focus was on treatment rather than diagnosis, we assumed the “active TB” compartment to be a weighted average of smear-positive and smear-negative pulmonary TB, thus avoiding the requirement to explicitly parameterize smear status. These simplifying procedures allowed us to generate a model with a minimum of parameters and assumptions, ensuring that model behavior was driven by the parameters of greatest interest and limiting the potential for results to be driven by extraneous factors.

We first set the rate at which individuals with active TB are diagnosed and initiate treatment (“TB treatment rate”) such that the duration of active TB matched the WHO-estimated duration of disease (prevalence/incidence), using the most recent data available at the time of the analysis (2012); at steady-state, this rate corresponded to 67% of active TB cases initiating treatment before death or spontaneous resolution, similar to WHO global estimates [1]. Using a modified downhill simplex approach, we then estimated a transmission parameter (number of secondary infections per infectious person-year) that resulted in the 2012 WHO-estimated global TB incidence at steady-state to within ±0.1. We used the steady-state model as our initial population, both for mathematical rigor and to improve the ability for others to replicate and generalize our results.

Other model parameters were taken as fixed, based on best available literature; parameters relating to TB mortality and treatment failure, default and success were based on WHO data (Table 2) [1]. Additional details on input derivation are provided in Appendix S1 in Table S2. Primary model outcomes are obtained using the reference values in Tables 1 and 2 as inputs.

**Table 2:** Selected key input parameters for estimating transmission impact of shorter TB regimens*.

Parameter	Reference value	Sensitivity analysis range	Reference(s)
**Baseline annual incidence (per 100,000 population)**	125	62–250	[1]
** Transmissions per person-year** †	8.5	6.8–20	[31]
** % infections progressing immediately to active TB** †	15%	5.0–21.0%	[23]
**Protection from reinfection w/prior infection**	60%	30–100%	[32]–[34]
**Relative infectiousness during treatment phase 1 (first 2 weeks) compared to active TB**	50%	0–100%	Assumed
**Annual risk of reactivation from latent to active TB**	0.05%	0.03–0.10%	[35],[36]
**Annual risk of relapse after completed treatment**	0.10%	0.05–0.20%	[37]
**Probability of failure among those who complete treatment**	2%	1–4%	[1]
**Life expectancy, years**	70	40–100	[38]
**Active TB mortality, per year**	20%	10–40%	[19]
**Self-cure without treatment, per year**	20%	10–40%	[19]
**Case detection ratio**	67%	62–70%	[1]

* Additional model parameters are listed in Table 1

† The transmission rate was initially calibrated to TB incidence. In sensitivity analyses, incidence was varied by varying one of these two parameters (both gave similar results); the two parameters were then also varied over the ranges listed, with the other parameter varied to maintain constant incidence.

### Sensitivity and uncertainty analyses

We performed wide sensitivity analyses on model data parameters to assess the robustness of our findings and their generalizability to alternative epidemiological settings. We selected upper and lower bounds for each parameter based on literature estimates (Tables 1, 2). For parameters that strongly influenced TB incidence (transmission rate, proportion of infections resulting in “primary progressive” TB, protection from reinfection in the latent TB state), we evaluated scenarios corresponding to 50–200% change from the baseline incidence. We therefore evaluated settings of “moderate” (62 per 100,000/year), “global reference” (125 per 100,000/year), “very high” (250 per 100,000/year), and “extreme” (1,000 per 100,000/year) incidence [1], by varying the transmission rate, primary progression and latent protection parameters individually. The modeled impact of shorter regimens on incidence remained similar regardless of which of these three parameters was varied. For simplicity, therefore, we present only results from varying the proportion of primary progression. Similarly, we evaluated the proportion of treatment default, which varies widely across settings, by constructing alternative scenarios of “low” (3%), “global reference” (7%), “high” (12.5%), and “very high” (25%) default. We assessed all possible combinations of incidence/default scenarios in a two-way sensitivity analysis.

In order to further assess the range of results that might be expected across a wide range of epidemic settings (in which parameter values would be expected to vary simultaneously), we performed a probabilistic uncertainty analysis using Latin Hypercube Sampling to generate at least 1,000 probabilistic combinations of values for all model parameters simultaneously [27]. Values for each parameter were sampled from beta distributions with the baseline value as the mode, upper and lower bounds of ±50% baseline, and shape parameter (alpha) of 4. We excluded simulations resulting in unrealistic scenarios for a globally representative epidemic (i.e., greater than ±50% variation in baseline incidence [62–188 per 100,000]) and verified that this did not result in a biased selection of individual parameters (Figure S3 in Appendix S1). Uncertainty ranges for model outcomes were calculated using the 2.5^th^ and 97.5^th^ percentiles of 1,000 simulations after restricting results in this fashion.

We also assessed the ability of our model to replicate the results of previous models of shorter TB treatment that did not consider the efficacy of partial treatment. We modified our model's transition parameters such that default always resulted in treatment failure (and return to the infectious active TB state), and we set the probability of treatment success upon completion of shorter regimens using data inputs from one such model (six-month regimen: 84%; four-month regimen: 89%; two-month regimen: 96%) [17]. Finally, we assessed the effect of changes in structural assumptions (details in Appendix S1). All simulations were performed using R, version 3.0.1 (R Foundation for Statistical Computing).

## Results

### Epidemiologic impact of shorter treatment regimens

**Figure 3:**
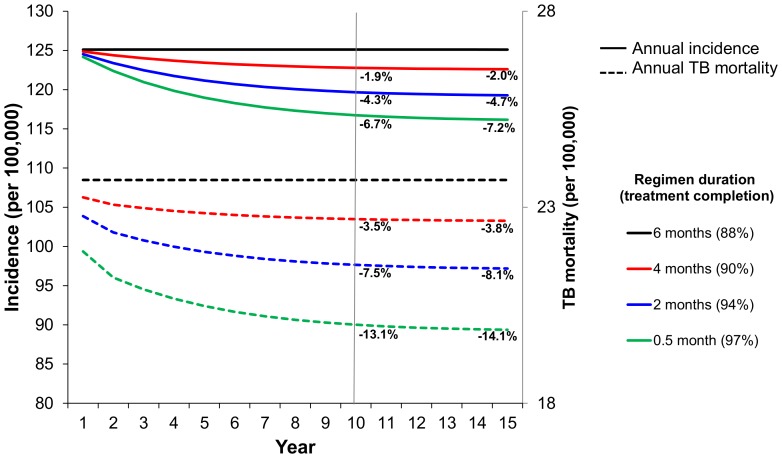
Reduction in TB incidence and mortality achievable from shorter-course regimens over time. Assuming TB incidence of 125 per 100,000/year, and 7% overall treatment default, the implementation of a four-month regimen vs. a six-month regimen results in a 1.9% reduction in incidence at 10 years (vertical line marks year 10 after introduction of a new regimen). Hypothetical two-month and two-week regimens decrease incidence by 4.3% and 6.7% respectively.

Primary model outcomes are shown in Figure 3. Starting from a steady-state “global reference” rate of 125 new cases per 100,000 population, introducing a four-month treatment regimen reduced incidence by only 1.9% [95% uncertainty range 0.6–3.1%] over 10 years; the shorter two-month and two-week regimens reduced incidence by 4.3% [1.8–7.0%] and 6.7% [3.0–10.2%], respectively. For all treatment durations, the rate of incidence reduction peaked in years 2–3, suggesting that the greatest impact of shorter TB regimens on transmission would occur within the first few years of implementation. The impact on TB mortality was greater but still modest. The four-month, two-month, and two-week regimens reduced mortality by 3.5%, 7.5%, and 13.1% at 10 years, respectively (Figure 3).

### Scenario analyses

We assessed the robustness of our findings to a variety of epidemic settings, reflecting the wide variations in disease transmission and treatment infrastructure across countries. Shortening the average duration of infectiousness before diagnosis from 16 to 2 months while maintaining the baseline incidence attenuated the impact of the four-month regimen (1.0% incidence reduction at 10 years). The impact of novel regimens on TB incidence was greater (2.4% 10-year reduction) in a very high-incidence scenario (250 per 100,000/year, similar to Ethiopia [1]) and attenuated (1.0% 10-year reduction) in a moderate-incidence scenario (62 per 100,000/year, similar to China [1]), reflecting the relative proportion of incident TB due to recent transmission in such settings. Effects on TB mortality were similar in both scenarios (3.2% [moderate incidence] – 3.7% [very high incidence] 10-year reduction). Finally, in the setting of low treatment default (3%), the four-month regimen decreased incidence by only 0.7% at 10 years, whereas in settings of high (12.5%) and very high (25%) default, incidence fell by 3.4% and 7.1%, respectively. To compare our findings with those of previous models, we constructed a scenario in which partial treatment was assumed to have no efficacy, with additional parameter changes as described in the Methods. This resulted in incidence reductions of 10.3% at 10 years and 10.5% at 35 years with a four-month regimen.

### Sensitivity analyses

**Figure 4:**
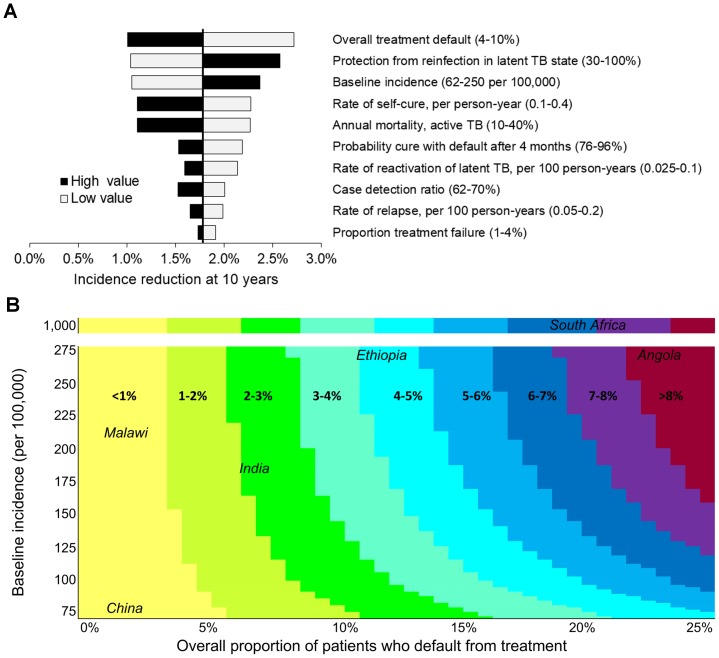
Sensitivity analyses. One-way and two-way sensitivity analyses of the difference in incidence at year 10 after introduction of a four-month regimen versus continuation of a six-month regimen of equal efficacy. A) One-way sensitivity analyses. Input parameters were varied one at a time within ranges consistent with estimates in the literature (Table 2). In this figure, we varied incidence by varying the transmission rate, but no major differences were observed when we instead varied the proportion of rapid progression to active disease. The parameters that most significantly influenced the impact of a four-month vs. six-month treatment regimen were the degree of protection afforded by latent infection, incidence of TB disease, and the proportion of treated patients who default at baseline. B) Two-way sensitivity analysis. The two most influential parameters likely to vary widely across epidemiological settings (TB disease incidence and proportion of treated patients defaulting at baseline) were varied simultaneously in a stepwise manner, within a range consistent with estimates in the literature and various epidemiologic settings (Table 2). Colors correspond to the range of projected incidence reduction for each combination of baseline incidence and treatment default and selected countries with representative estimates are shown. The highest estimates for both treatment default (25%) and baseline incidence (1,000 per 100,000/year) resulted in no more than 8.3% incidence reduction with a four-month vs. six-month regimen at 10 years.

In one-way sensitivity analyses, no scenario resulted in an incidence decrease of more than 2.7% at 10 years with four-month therapy (Figure 4a). Other than the protection afforded by latent infection, the two most influential parameters were the baseline TB incidence and the treatment default proportion. We therefore conducted a two-way sensitivity analysis on these parameters; the most extreme combination (incidence 1,000 per 100,000; 25% default) led to 8.3% incidence reduction at 10 years with four-month therapy (Figure 4b). In a moderate-incidence setting (100 per 100,000/year) with a well-functioning TB control program (3% default at six months), the four-month regimen was projected to reduce incidence by 0.6% [95% uncertainty range 0.1–1.1%] at 10 years, whereas in a very high-incidence scenario (300 per 100,000/year) with poor follow-up (20% default) incidence decreased by 7.2% [3.0–11.6%]. Even in the high-burden scenario, the uncertainty analysis yielded incidence reductions of ≥10% in only 8.5% of simulations.

## Discussion

This mathematical model of TB treatment and transmission suggests that novel treatment regimens are unlikely to have the dramatic impact on global TB incidence projected by earlier models; specifically, we found that immediate implementation of a four-month treatment regimen could reduce TB incidence by 1.9% and mortality by 3.5% over 10 years compared to a six-month regimen of equal efficacy, suggesting that previous analyses significantly overestimated the impact of shortened treatment duration. The impact of novel shorter-course TB regimens is likely to be greater in high-incidence, high-default settings, but in most settings these regimens should be recommended on the basis of their clinical effectiveness and potential cost-effectiveness rather than a large projected impact on population-level incidence and transmission.

As with all modeling analyses, we made assumptions about structure (e.g., uninfected, latent, active TB compartments), parameter values, and transmission dynamics (e.g., homogeneous mixing). However, we selected a model that would minimize extraneous assumptions, in order to clearly demonstrate relationships between input parameters and outputs. We also varied data parameters and structural assumptions to explore a wide range of natural history, treatment, and epidemiological scenarios, with no significant change in our findings. Our results suggest more modest benefits compared to prior analyses that modeled the impact of shorter regimens by increasing the total proportion of patients completing treatment while implicitly assuming no effectiveness of partial treatment (even up to 5.9 months of a six-month treatment course completed). When we likewise assumed that partial treatment had zero efficacy, we were able to replicate the findings of an earlier model [17] with our simpler, more transparent framework (10.5% [current model] vs. 10% [prior model] incidence reduction at 35 years with a four-month regimen). This suggests that the difference in projected epidemiological impact between previous analyses and the present model is attributable not to differences in the structure or parameter values of the two models, but rather to our incorporation of partial treatment efficacy [17].

In our model, even a two-week regimen resulted in an incidence reduction of only 6.7% at 10 years. However, if TB treatment could be made so short and non-toxic (similar to many typical antibiotic regimens) that clinicians were willing to prescribe it empirically, without waiting for diagnostic confirmation, such regimens might reduce transmission by removing delays and barriers to treatment after diagnosis; these ancillary benefits of shorter-course therapy are not incorporated in our model and may lead to underestimation of the true impact of new regimens. This underestimation is likely to be greater for ultra-short-course regimens (e.g., two weeks) than for regimens (e.g., four months) that may not be perceived as qualitatively shorter than current treatment. Because our estimates of partial treatment efficacy relied on clinical trials of regimens that are similar to the currently recommended first-line regimen, they may not reflect the efficacy of future regimens that will likely include new classes of drugs. Still, our findings remained robust to wide variations around the partial efficacy parameters in sensitivity analyses. It is important to note that novel treatment regimens are expected to provide benefits in terms of patient satisfaction, cost-effectiveness, and increased barrier to drug resistance, and should thus remain a high research priority. However, the primary justification for deploying these regimens should be that they are beneficial to patients and health systems, not the expectation of significant impact on transmission.

Limitations of this analysis include the simplicity of the model; the model was based on global TB epidemic data and therefore may not generalize to unique epidemiological settings (e.g., prisons and other areas of high drug resistance) or settings of lower TB incidence. We intentionally chose a simple approach in order to generate a transparent modeling framework that could demonstrate the transmission impact of novel regimens in a population that is generalizable, through sensitivity analysis, to a number of potential epidemiologic settings. Nevertheless, our results are not precisely calibrated to any single population, and our sensitivity analyses suggest that the effect of shorter treatment duration on population-level incidence may vary considerably depending on the epidemic setting, with the most important drivers of impact being TB incidence and treatment default proportion. Although our results remained robust in a wide range of sensitivity analyses, our estimation of global average reductions in incidence may not reflect the likely greater impact of shorter regimens in settings of very high incidence and very high treatment default, nor do they take into account co-dynamics with HIV. It will therefore be important to conduct further analyses with models that are closely calibrated to unique epidemic and health system resource settings, particularly those (e.g., Southern Africa) with the highest rates of both TB incidence and HIV/TB co-infection.

In summary, we have used a simple, generalizable modeling framework, populated by data from randomized trials, to demonstrate that novel shorter-course TB treatment regimens are unlikely to reduce incidence by more than 3% (upper bound of uncertainty range for a four-month regimen) to 7% (two-month regimen) over 10 years in most epidemiological settings. The projection of greater impact by previous models appears to reflect the assumption that TB therapy confers no benefit until the entire course is complete. Future studies should assess the benefits of novel regimens in specific settings with high TB incidence, treatment default, and TB-HIV co-infection, as these settings are where novel first-line regimens may have the most impact. While awaiting the results of such studies, novel TB regimens should be prioritized based on their ability to improve individual clinical outcomes and provide potential benefits to an overburdened healthcare system, not the expectation that they will dramatically reduce TB incidence and mortality at the population level.

## Supporting Information

Appendix S1 **This file includes Tables S1 to S2 and Figure S1 to S3.** Table S1: Initial state conditions Table S2: Model parameters Figure S1: Model structure, including parameter definitions Table S3: Additional sensitivity analysis results Figure S2: Structural sensitivity analyses on (A) latent infection and (B) age structure. Figure S3: Distribution of input values and incidence in uncertainty analysis.
(PDF)
