# Supplementary material for: Population-Level Impact of Shorter-Course Regimens for Tuberculosis: A Model-Based Analysis
Source: PLoS One. 2014 May 9;9(5):e96389. doi: 10.1371/journal.pone.0096389 (PMC4015982; doi:10.1371/journal.pone.0096389)
Supplement: Appendix S1 — This file includes Tables S1 to S2 and Figure S1 to S3. Table S1: Initial state conditions Table S2: Model parameters Figure S1: Model structure, including parameter definitions Table S3: Additional sensitivity analysis results Figure S2: Structural sensitivity analyses on (A) latent infection and (B) age structure. Figure S3: Distribution of input values and incidence in uncertainty analysis. (PDF) [file pone.0096389.s001.pdf]

Population-level impact of shorter-course regimens for tuberculosis: a model-based analysis

Appendix S1

Table S1: Initial state conditions

State	Description	Number of individuals, per 100,000
S(0)	# in Susceptible compartment	62,280
L(0)	# in Latent TB compartment	31,197
A(0)	# in Active TB compartment	104
$T_1(0)$	# in Treatment phase 1 compartment	4
$T_2(0)$	# in Treatment phase 2 compartment	11
$T_3(0)$	# in Treatment phase 3 compartment	14
$T_4(0)$	# in Treatment phase 4 compartment	13
C(0)	# in Cured compartment	6,377
N	Total population	100,000

Table S2: Model parameters

Parameter	Description	Reference/baseline value [95% uncertainty range]	Ref(s)
β	Transmission rate per person-year	8.5 [4.25-12.75]	[1]
p	Relative susceptibility to infection after prior exposure to TB	0.4 [0.20-0.60]	[2,3]
ri	Relative infectiousness in treatment phase 1 compared to active TB	0.5 [0.25-0.75]	
r	Proportion of infections progressing immediately to active TB	0.15 [0.09-0.26]	[4]
er	Rate of endogenous reactivation from latent to active TB, per year	0.0005 [0.0003-0.0008]	[5,6]
sc	Self-cure rate, per year	0.2 [0.10-0.30]	[7]
μ_0	Background mortality rate, per year	0.014 [0.01-0.03]	[8]
μ_{TB}	Mortality rate in active TB state, per year	0.2 [0.10-0.30]	[7]
rl	Rate of relapse after treatment, per year	0.001 [0.0005-0.0015]	[9]
cd	TB treatment rate, per year	0.84 [0.42-1.26]	[10]
d	Overall proportion of treatment default	0.07	[10]
m	Overall proportion of deaths on treatment	0.04	[10]
f	Overall proportion of treatment failure	0.02	[10]

Table S2 (cont.)

Parameter	Description	Reference/baseline value [95% uncertainty range]				Ref (s)
		i= 1	***i= 2***	***i= 3***	***i=4***	
t_i	Duration of treatment phase *i*, in years	1/24	1/8	1/6	1/6	
d_i	Proportion of all defaults from a 6-month regimen that occur in phase *i*	0.03	0.27	0.38	0.32	[11]
δ_i	Treatment default rate in phase *i*, per year: $d*d_i/t_i$	0.05 [0.03-0.08]	0.15 [0.08-0.23]	0.16 [0.08-0.24]	0.13 [0.07-0.20]	[10]
m_i	Proportion of all deaths on a 6-month treatment regimen that occur in phase *i*	0.27	0.32	0.205	0.205	[12-14]
μ_i	Total (background + TB-specific) mortality rate in phase *i*, per year: $m*m_i/t_i$	0.26 [0.13-0.39]	0.10 [0.05-0.15]	0.05 [0.02-0.07]	0.05 [0.02-0.07]	[7]
tf_i	Treatment failure rate in phase *i* for regimen with *n* treatment phases, per year (for $i=n$): f/t_i	0.48 [0.24-0.72]	0.16 [0.08-0.24]	0.12 [0.06-0.18]	0.12 [0.06-0.18]	[10]
	(for $i<n$)	0	0	0	0	
tc_i	Rate of continuation from phase *i* to phase *i+1* (or to cure if $i=n$), per year: $1/t_i - (tf_i+\delta_i+\mu_i)$	23.69	7.75	5.79	5.70	
dfc_i	Proportion in a 6-month regimen who are cured after default in phase *i*	0	0.16 [0.08-0.23]	0.63 [0.31-0.94]	0.86 [0.43-1]	[10]
dff_i	Proportion in a 6-month regimen who return to active TB after default in phase *i*	1	0.84 [0.77-0.92]	0.38 [0.06-0.69]	0.14 [0-0.57]	[15,16]

Model inputs

We derive our model inputs from published data on the natural history and treatment outcomes of TB. In order to account for the four phases of treatment of varying duration in our model, we convert proportions to time-dependent rates by dividing proportion values by the duration (in years) of each treatment phase. Take, for example, treatment phase 1, which lasts two weeks (t_1 = 1/24 of a year). Thus, the sum of all exit rates from this phase (failure, tf_1, phase completion, tc_1, mortality, μ_1 and default, δ_1) should equal 24/year. We next calculate the proportion of individuals entering phase 1 who exit by each of these four routes; for example, the proportion of individuals who die is calculated as the overall proportion of individuals who die (m = 0.04), multiplied by the proportion of those deaths that occur in phase 1 (m_1 = 0.27). The proportion of individuals who default is calculated in similar fashion. The proportion of individuals who fail is assumed to be zero unless the regimen ends at the end of the phase (i.e., in phase 1, the failure proportion is zero for two-month, four-month, and six-month regimens), in which case the failure proportion is assumed to be a value that is the same for all regimens (f = 0.02 at baseline). Thus, f represents the probability of failure, conditional on completing therapy.

Figure S1: Model structure, including parameter definitions

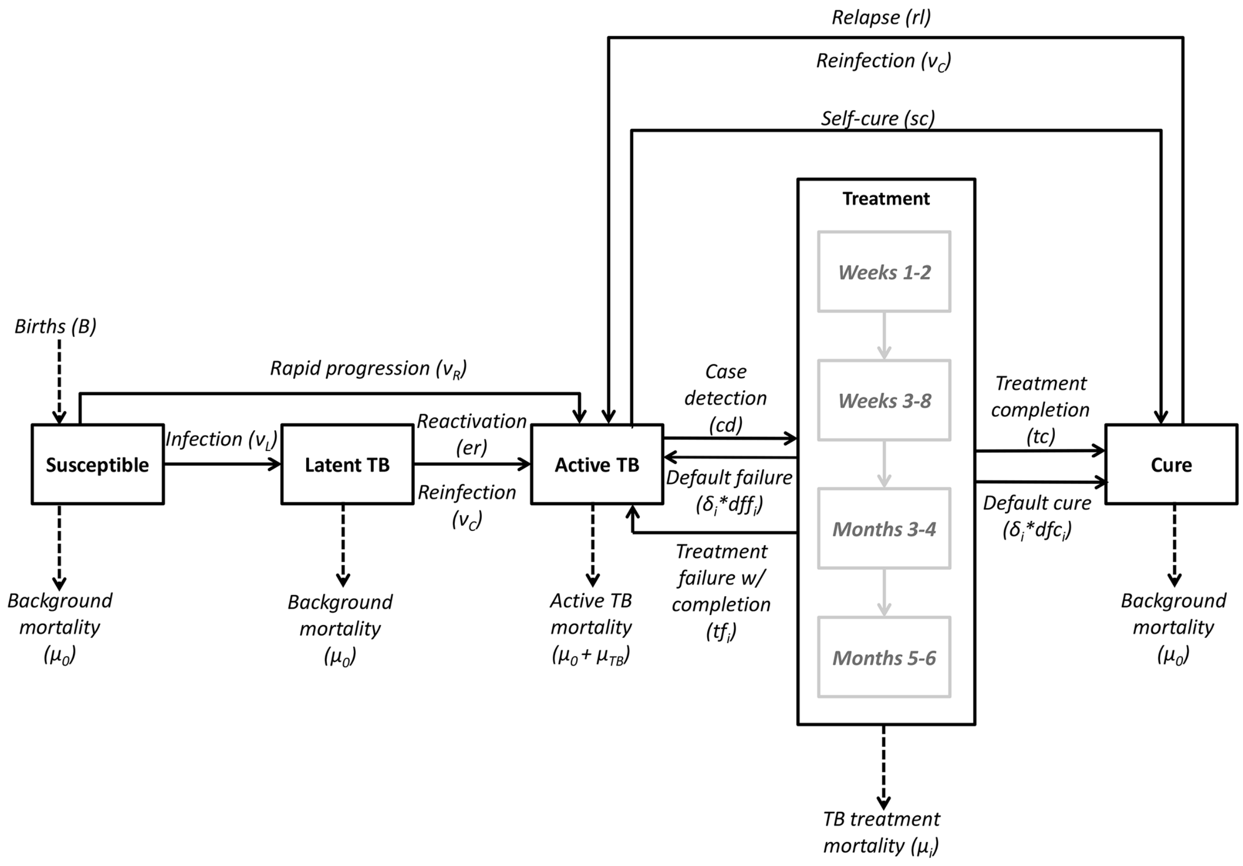

Model equations

Figure S1 shows a schematic representation of the model with relevant equations and rate constants for transitions between compartments. Initial state conditions for each compartment are listed in Table S1. Model parameters, reference values, and the range of values used in the probabilistic uncertainty analysis are listed in Table S2.

Force of infection

$$F(t)=\frac{\beta+(ri\cdot T_1(t))}{N}$$

The rate at which individuals in each compartment become infected with TB depends upon the force of infection (probability of an uninfected individual becoming infected per unit time), which varies with time according to the number of infectious (i.e., in active TB or treatment phase 1) individuals in the population. Thus, the force of infection $F(t)$ is the product of the number of transmissions per unit time β¸ the number of individuals with active TB $A(t)$, the number of individuals in the first treatment phase $T_1(t)$ multiplied by the relative infectiousness of that compartment *ri*, divided by the total number of individuals in the population *N* (held constant at 100,000 in this model). We assume homogenous mixing of the population, such that each susceptible individual has an equal chance of coming into contact with an infectious individual.

Infection with rapid progression to active TB

$$\nu_R(t)=F(t)\cdot r$$

Infection with latent TB

$$\nu_L(t)=F(t)\cdot(1-r)$$

A proportion (*r*) of individuals who become infected with TB progress immediately to active disease, such that the rate of progression from the susceptible state to active disease $v_R(t)$ is equal to the force of infection $F(t)$ multiplied by the proportion of rapidly progressing infections *r*. The remainder (*1-r*) of newly infected individuals progress at a rate $v_L(t)$ to a state of latent disease, in which they are not infectious.

Reinfection from latent or cured state, with rapid progression to active TB

$\nu_C(t)=F(t)\cdot r\cdot p$

Individuals in the latent and cured states can become reinfected with TB at a rate determined by the force of infection *F(t)* and the proportion (*r*) progressing immediately to active disease. Prior exposure to TB confers relative protection against reinfection, such that only a proportion *p* of individuals in the latent or cured states are susceptible to infection. We vary this “latent protection” parameter widely in sensitivity and uncertainty analyses.

Births

$$B(t)=\mu_0(S(t)+L(t)+C(t))+(\mu_0+\mu_{TB})A(t)+\sum_{i=1}^{4}\mu_i\cdot T_i(t)$$

We model a closed population, with the number of births *B(t)* equivalent to the number of deaths at any time. The mortality rate constants for each compartment are listed in Table S2. Individuals in the susceptible, latent and cured states [*S(t)*, *L(t)*, and *C(t)*, respectively] progress to death according to a background mortality rate μ_0 based on a life expectancy at birth of 70 years [8]. Individuals in the active TB state *A(t)* have an additional, TB-related mortality risk μ_{TB}. Individuals in the treatment compartments are subject to a mortality rate μ_i that varies with each treatment phase T_i, with $1\leq i\leq 4$.

Relapse, reactivation, treatment initiation, self-cure

We assume constant rates of relapse after treatment and reactivation of latent TB to active disease (see Table S2). Individuals in active disease are detected and diagnosed at a constant rate

to progress to the first phase of treatment. They may also experience self-cure and progress to the cured state without going through treatment.

Treatment

We model varying durations of treatment by determining the number of treatment phases (*n*) required for a full treatment course in each simulation, such that the six-month regimen requires completion of $n = 4$ phases and the four-month regimen requires completion of $n = 3$ phases, etc... Entry into the "Cure" compartment requires either completion of treatment phase *n* or cure after default from any treatment phase 1 through *n*. Individuals may complete each phase of treatment, die or default. A proportion of defaulters (*dfc*) will have undergone sufficient treatment to progress to cure, while the remainder (*dff*) returns to the active TB state.

The differential equations describing rates of transition between model compartments are as follows:

- ***Susceptible, S***: $\frac{dS}{dt} = B(t) - S(t) \cdot (\nu_R(t) + \nu_L(t) + \mu_0)$

  At any time, entry into the susceptible state is determined by the number of births *B(t)*. The rate of exit is determined by the sum of the rates of progression to active disease, latent disease, and mortality ($\nu_R(t)$, $\nu_L(t)$, and μ_0, respectively).
- ***Latent, L***: $\frac{dL}{dt} = (S(t) \cdot \nu_L(t)) - L(t) \cdot (er + \nu_C(t) + \mu_0)$

  At any time, the number of new latent infections is determined by the number of susceptible individuals *S(t)* multiplied by the rate of progression to latent disease $\nu_L(t)$. The rate of exit is determined by the number of individuals in the latent TB compartment *L(t)* multiplied by the

sum of the rates of endogenous reactivation to active disease *er*, reinfection with rapid progression to active disease $v_C(t)$, and mortality μ_0.

- ***Active TB, A***: $\frac{dA}{dt} = (S(t)\cdot\nu_R(t)) + L(t)\cdot(er + \nu_C(t)) + C(t)\cdot(rl + \nu_C(t)) + \sum_{i=1}^{n}[T_i(t)\cdot[(\delta_i\cdot dff_i) + tf_i]] - A(t)\cdot(\mu_0 + \mu_{TB} + cd + sc)$

At any time, the number of new cases of active disease includes cases resulting from rapid progression after initial infection at rate $v_R(t)$for individuals in the susceptible state $S(t)$, endogenous reactivation at rate *er* and reinfection at rate $v_C(t)$ for individuals in the latent state $L(t)$, and relapse at rate *rl* and reinfection at rate $v_C(t)$ for individuals in the cured state, in addition to failure after treatment completion at rate tf_i and the proportion dff_i of individuals in each treatment phase $T_i(t)$ who default at rate δ_i and subsequently return to the active disease state. The rate of exit is determined by the sum of the rates of background mortality μ_0, TB-specific mortality μ_{TB}, TB treatment rate *cd*, and self-cure without treatment *sc*.

- ***Treatment phase 1, T₁***: $\frac{dT_1}{dt} = (A(t)\cdot cd) - T_1(t)\cdot(tc_1 + \delta_1 + tf_1 + \mu_1)$
- ***Treatment phase 2, T₂***: $\frac{dT_2}{dt} = (T_1(t)\cdot tc_1) - T_2(t)\cdot(tc_2 + \delta_2 + tf_2 + \mu_2)$
- ***Treatment phase 3, T₃***: $\frac{dT_3}{dt} = (T_2(t)\cdot tc_2) - T_3(t)\cdot(tc_3 + \delta_3 + tf_3 + \mu_3)$
- ***Treatment phase 4, T₄***: $\frac{dT_4}{dt} = (T_3(t)\cdot tc_3) - T_4(t)\cdot(tc_4 + \delta_4 + tf_4 + \mu_4)$

At any time, entry into the first phase of treatment is determined by the rate of detection *cd* for individuals in the active disease state $A(t)$. The number of individuals from each phase of treatment *i* who enter the subsequent phase of treatment $i+1$ (or the cured state if phase *i* is the last phase of treatment) is equal to the rate of treatment continuation tc_i multiplied by the

number of individuals in each treatment phase $T_i(t)$. Individuals may exit each treatment phase by death at rate μ_i, default at rate δ_i, continuation to the next phase at rate tc_i, and failure of treatment at rate *tfi* if they are in the final phase.

- ***Cure, C***: $\frac{dC}{dt} = (A(t) \cdot sc) + (T_n(t) \cdot tc_n) + \sum_{i=1}^{n} (T_i(t) \cdot \delta_i \cdot dfc_i) - C(t) \cdot (rl + \nu_C(t) + \mu_0)$,

where n = index for last phase of treatment in regimen (e.g., n = 4 for six-month regimen, n = 2 for two-month regimen).

At any time, the number of new cured cases includes self-cure at rate *sc* for $A(t)$ individuals in the active disease state, treatment success at rate tc_n for $T_n(t)$ individuals in the last treatment phase, in addition to the proportion dfc_i of the $T_i(t)$ individuals in each treatment phase who experience default at rate δ_i and subsequently progress to the cured state. Exit from the cured state is determined by the rates of relapse *rl*, reinfection $\nu_C(t)$, and mortality μ_0.

Partial treatment efficacy data inputs

We estimated the proportion of relapse among those completing one-third, one-half or the entirety of the treatment regimen based on relapse outcomes in early clinical trials of short-course TB regimens [15,17]. These trials report 24% and 14% relapse after 60 months of follow-up for two-month and four-month treatment regimens consisting of streptomycin, isoniazid, rifampin, and pyrazinamide. Similar outcomes were achieved with a four-month regimen in a trial conducted in East Africa [18]. We estimate the probability of stable cure for patients who default after two months or four months of treatment based on the proportion of patients who did not experience relapse over long-term follow-up in these trials. As a conservative approach, we assume a “stepwise” distribution of the probability of cure after default in each phase of

treatment; for example, the probability of cure with default at any point between four and six months is estimated as the probability of cure with completion of four months of treatment.

The best estimate that we found for the probability of cure after two months of treatment was from a clinical trial of a two-month regimen in patients with smear-negative, culture-positive pulmonary tuberculosis [15]. We used data from a review of early clinical trials of first-line TB regimens of two to six months to estimate a correction factor based on the assumption of relatively faster progression to cure among smear-negatives, who are thought to have a lower bacillary burden, compared to smear-positive TB patients [16,19]. Because these trials were conducted in the 1970s, we presume that HIV was not a significant factor in the outcomes of smear-positive vs. smear-negative TB. We estimated that the probability of relapse after two months of treatment is twice as high among smear-positive than smear-negative cases; in sensitivity analyses, we vary this correction factor from 1 to 3 and further vary the probability of cure with 2 months of treatment by ±50% to account for the uncertainty in the values of these parameters. We then compute a weighted probability of cure using the relative prevalence of smear-negatives and smear-positives among new TB cases [10].

We used interpolation to derive estimates for the probability of cure after two weeks of treatment, assuming a linear increase between initiation of treatment and the completion of two months. This results in an estimated probability of cure of 15.6% among individuals who complete two to eight weeks of treatment. To account for the scarcity of data for these estimates and the inherent uncertainty related to our assumptions, we used a wide range of estimates in

sensitivity analyses around these parameters. We assumed that there is no chance of cure with default during the first two weeks of treatment.

For the shortened treatment regimens, we adjusted these estimates of probability of cure after default as follows: for each 1/3 incremental reduction in total treatment duration from the six-month regimen (to four months and two months), the probability of cure at phase *n* increases by 1/3 of the difference in probability of cure between phases *i* and *i*+1 in the six-month regimen. For example, given probabilities of cure of 16%, 63%, and 86% at two weeks, two months and four months in the six-month regimen, the probability of cure at two months in the four-month regimen is computed as 63% + 1/3 * (86% - 63%) = 70%. In sensitivity analyses, we vary this correction factor for the probability of cure (*cpcf*) from 1/6 to 1/2.

We also assess the impact of using linear interpolation of the clinical trial data to set the probability of cure with default. This represents a less conservative approach, with higher probabilities of cure compared to using the stepwise distribution described above. For instance, the probability of cure for those who default between months 2 and 4 is computed as the mean of the proportions cured with two-month and four-month courses of treatment in the trials, rather than as the proportion cured with the two-month treatment course. This results in probabilities of cure of 31%, 74%, and 92% with default in treatment phases 2, 3, and 4 of standard six-month therapy (vs. 16%, 63%, and 86% with stepwise distribution) but does not significantly alter results on the transmission impact of novel regimens (1.3% incidence reduction at 10 years with four-month vs. six-month regimen compared to 1.9% with stepwise distribution).

Structural sensitivity analyses

As a sensitivity analysis, we repeated the main analysis in a setting of declining incidence rather than a steady state. We first initialized the model at a steady state reflective of the TB epidemic a decade ago, then reduced the transmission rate and simulated the course of the epidemic to obtain a decline reflective of current global TB incidence estimates. We then simulated the continuation of the 6-month regimen or the introduction of shorter regimens.

We also assessed the robustness of our findings to increased detail in model structure, by repeating the analysis with two alternate models. In Model 2, we replaced the single "Latent TB" compartment with three sequential compartments reflecting "Immediate" (year 1), "Recent" (years 2-5), and "Remote" (years 6 and beyond) latent infection, resulting in a total of 10 compartments (Figure S2A). In this model, reinfection can occur in the "Recent" and "Remote" latent infection compartments in addition to the "Cured" compartment, and results in return to the "Latent Immediate" compartment. Rapid progression can occur from either "Immediate" or "Recent" latent infection, resulting in transition to the "active TB" compartment. As in the primary model, we set the total proportion of latent infection of cases progressing rapidly to active TB at 15%, with 63% occurring in the first year and 37% occurring in the subsequent four years [3].

In Model 3, we replicated the structure of the original model four times to create four age subdivisions (0-14, 15-29, 30-44, and ≥45 years old), resulting in a total of 32 compartments (Figure S2B). Rates of background mortality for each age group are derived from global life tables estimates [8]. As in the original model, we maintained the total population constant by

setting the number of births in each timestep equal to the total number of deaths, with all individuals being born in the susceptible state in the 0-14 age subdivision. In addition to progressing through the TB states, individuals in the first three age subdivisions progress to the next age subdivision at a rate of 1/15 yr^{-1}. We used this model to replicate the analysis under varying assumptions of (1) equal rates of infection and reactivation across all age subdivisions, (2) rates of infection set to 50% of the baseline value among children (ages 0-14), and (3) rates of reactivation set to 50% of the baseline value among children to assess the impact of differential disease progression by age.

For both Model 2 and Model 3, we initialized the model at steady-state and projected incidence using the same procedures as in the primary analysis. The conclusions remained largely unchanged in all of these sensitivity analyses, with the reduction in incidence at 10 years with a 4-month vs. 6-month regimen ranging from 0.9% to 2.5% when taking into account the efficacy of partial treatment; as in the primary analysis, incidence reduction was overestimated when we did not account for this partial efficacy (5.1% to 13.5%; 5.3 to 5.7-fold). Detailed results are presented in Table S3.

Table S3: Additional sensitivity analysis results

	**Incidence reduction at 10 years**	
	With partial efficacy	***No partial efficacy***
Primary analysis	1.9%	10.3%
Declining incidence	1.7%	9.3%
Model 2 (latent infection)	0.9%	5.1%
Model 3 (age structure)	2.4%	12.8%
Model 3, differential infection rates	2.4%	13.0%
Model 3, differential reactivation rates	2.5%	13.5%

Figure S2: Structural sensitivity analyses on (A) latent infection and (B) age structure. Model parameters are the same as in the primary model except where indicated otherwise in the legend. Illustration of births and deaths in panel A and age progression in panel B are omitted for clarity.

A)

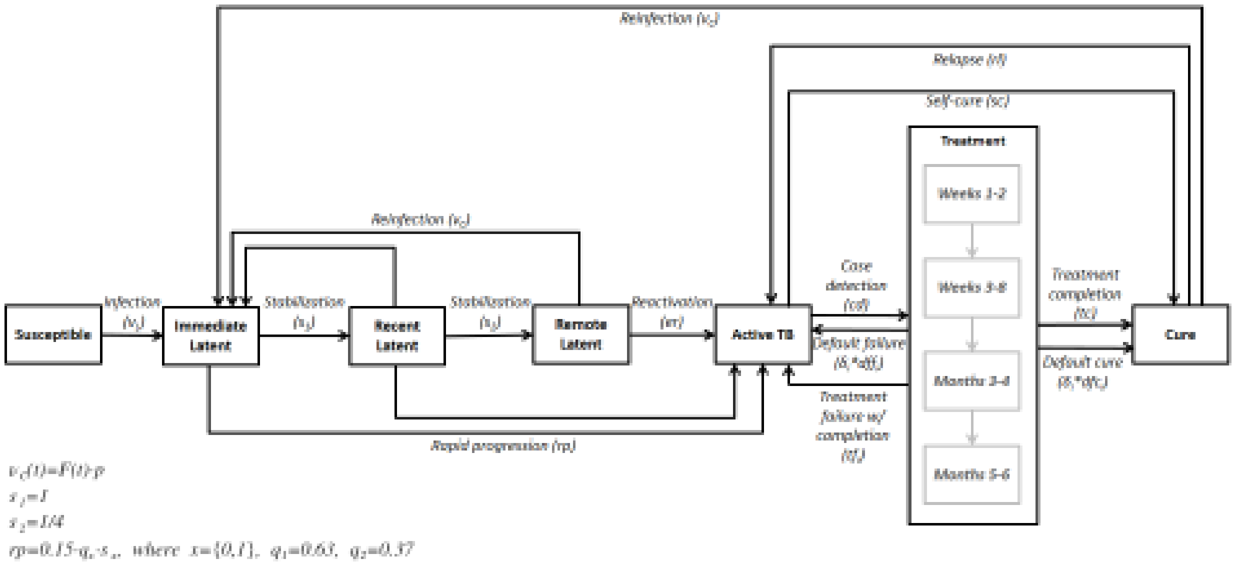

B)

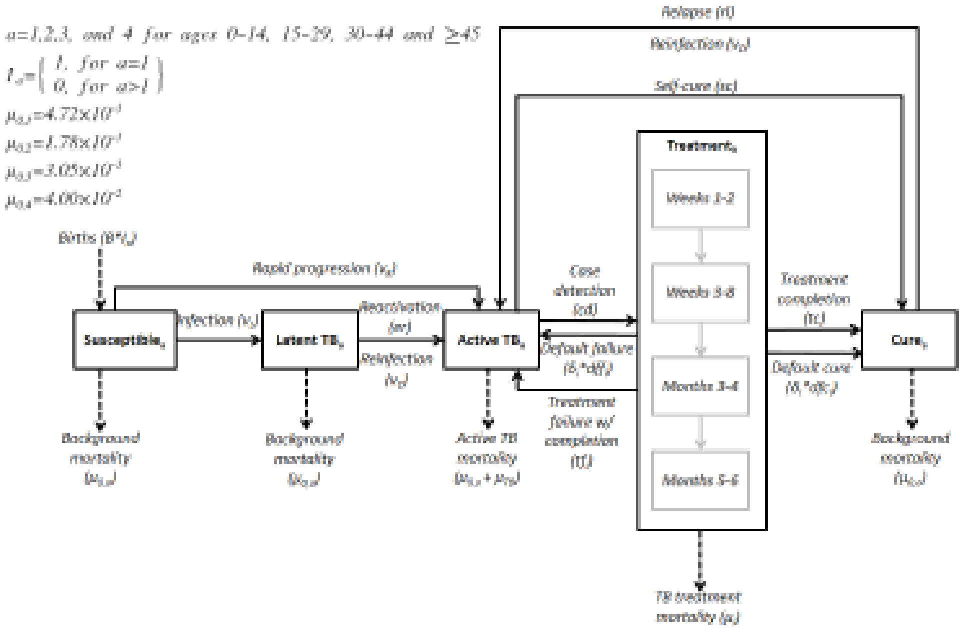

Uncertainty analysis

A total of 2,449 combinations of input values were generated using Latin Hypercube Sampling [20], of which 1,449 were excluded because they resulted in baseline incidence below or above the specified range (62-188 per 100,000). Incidence values from the remaining 1,000 combinations of inputs were used to generate 95% uncertainty ranges. This selection procedure did not induce appreciable bias in the range of selected values for any of the model parameters (Figure S3). We conducted a similar uncertainty range analysis for a moderate-burden setting (incidence 100 per 100,000 ±50% and 3% default proportion) and a high-burden setting (incidence 300 per 100,000 ±50% and 20% default proportion). A total of 2,086 and 4,009 combinations of input values were generated for the moderate-burden and high-burden settings respectively, of which 1,000 resulted in incidence values within the specified ranges and were used to generate the 95% uncertainty ranges.

Figure S3

A) Distribution of input values for each parameter used in uncertainty analysis and baseline incidence for each combination of input parameters

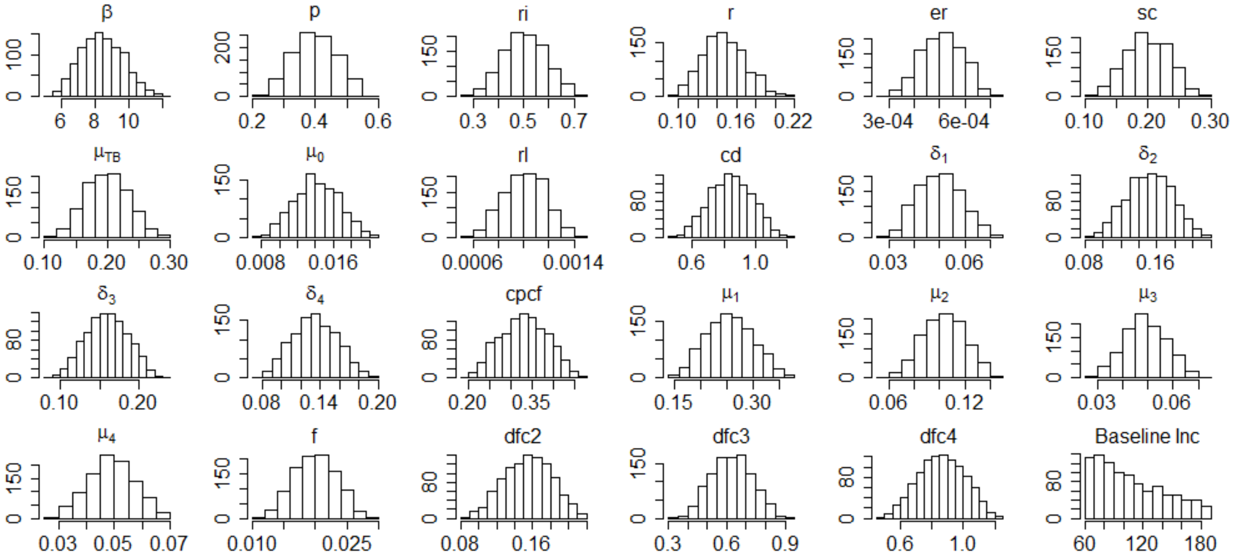

B) Distribution of incidence at 15 years with 6-month regimen with full set of initial input values generated by Latin Hypercube sampling (LHS) vs. restricted set (incidence 62-188 per 100,000)

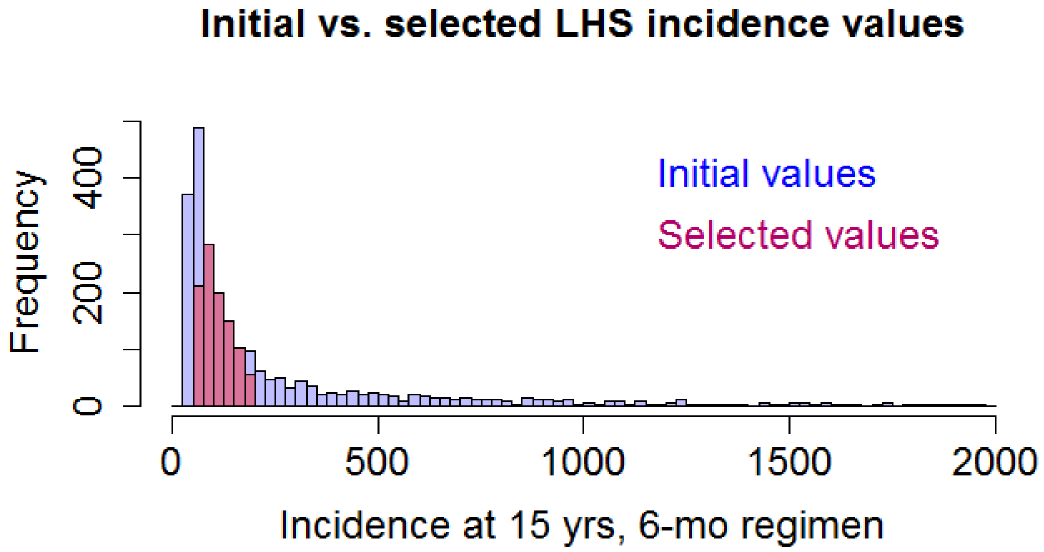

References

1. van Leth F, van der Werf MJ, Borgdorff MW (2008) Prevalence of tuberculous infection and incidence of tuberculosis: A re-assessment of the Styblo rule. Bull World Health Organ 86: 20-26.

2. Sutherland I, Svandova E, Radhakrishna S (1982) The development of clinical tuberculosis following infection with tubercle bacilli. 1. A theoretical model for the development of clinical tuberculosis following infection, linking from data on the risk of tuberculous infection and the incidence of clinical tuberculosis in the Netherlands. Tubercle 63: 255-268.

3. Vynnycky E, Fine PE (1997) The natural history of tuberculosis: The implications of age-dependent risks of disease and the role of reinfection. Epidemiol Infect 119: 183-201.

4. Blower SM, McLean AR, Porco TC, Small PM, Hopewell PC, et al (1995) The intrinsic transmission dynamics of tuberculosis epidemics. Nat Med 1: 815-821.

5. Harries AD, Dye C (2006) Tuberculosis. Ann Trop Med Parasitol 100: 415-431.

6. Horsburgh CR,Jr, O'Donnell M, Chamblee S, Moreland JL, Johnson J, et al (2010) Revisiting rates of reactivation tuberculosis: A population-based approach. Am J Respir Crit Care Med 182: 420-425.

7. Tiemersma EW, van der Werf MJ, Borgdorff MW, Williams BG, Nagelkerke NJ (2011) Natural history of tuberculosis: Duration and fatality of untreated pulmonary tuberculosis in HIV negative patients: A systematic review. PLoS One 6: e17601.

8. United Nations Population Division (2013) World population prospects: The 2012 revision. New York: United Nations.

9. Johnson JL, Hadad DJ, Dietze R, Maciel EL, Sewali B, et al (2009) Shortening treatment in adults with noncavitary tuberculosis and 2-month culture conversion. Am J Respir Crit Care Med 180: 558-563.

10. World Health Organization (2012) Global tuberculosis control 2012. Geneva: WHO.

11. Kruk ME, Schwalbe NR, Aguiar CA (2008) Timing of default from tuberculosis treatment: A systematic review. Trop Med Int Health 13: 703-712.

12. Harries AD, Hargreaves NJ, Gausi F, Kwanjana JH, Salaniponi FM (2001) High early death rate in tuberculosis patients in Malawi. Int J Tuberc Lung Dis 5: 1000-1005.

13. Jonnalagada S, Harries AD, Zachariah R, Satyanarayana S, Tetali S, et al (2011) The timing of death in patients with tuberculosis who die during anti-tuberculosis treatment in Andhra Pradesh, South India. BMC Public Health 11: 921.

14. Moolphate S, Aung MN, Nampaisan O, Nedsuwan S, Kantipong P, et al (2011) Time of highest tuberculosis death risk and associated factors: An observation of 12 years in Northern Thailand. Int J Gen Med 4: 181-190.

15. [Anonymous] (1984) A controlled trial of 2-month, 3-month, and 12-month regimens of chemotherapy for sputum-smear-negative pulmonary tuberculosis. Results at 60 months. Am Rev Respir Dis 130: 23-28.

16. Fox W (1981) Whither short-course chemotherapy? Br J Dis Chest 75: 331-357.

17. [Anonymous] (1986) Long-term follow-up of a clinical trial of six-month and four-month regimens of chemotherapy in the treatment of pulmonary tuberculosis. Singapore tuberculosis service/British medical research council. Am Rev Respir Dis 133: 779-783.

18. Aluoch JA (1978) Fourth study of short term chemotherapy by the African and British medical research councils. Bull Int Union Tuberc 53: 256-257.

19. Dutt AK, Stead WW (1994) Smear-negative pulmonary tuberculosis. Semin Respir Infect 9: 113-119.

20. Blower SM, Dowlatabadi H (1994) Sensitivity and uncertainty analysis of complex models of disease transmission: An HIV model, as an example. International Statistical Review / Revue Internationale de Statistique 62: 229-243.
